# No major association between TGFBR1*6A and prostate cancer

**DOI:** 10.1186/1471-2156-5-28

**Published:** 2004-09-22

**Authors:** Virginia Kaklamani, Lisa Baddi, Diana Rosman, Junjian Liu, Nathan Ellis, Carole Oddoux, Harry Ostrer, Yu Chen, Habibul Ahsan, Kenneth Offit, Boris Pasche

**Affiliations:** 1 Cancer Genetics Program, Division of Hematology/Oncology, Department of Medicine and Robert H. Lurie Comprehensive Cancer Center, Feinberg School of Medicine, Northwestern University, Chicago, Illinois, USA; 2 Clinical Genetics Service, Memorial Sloan-Kettering Cancer Center, New York, New York, USA; 3 Human Genetics Program, Department of Pediatrics, New York University Medical Center, New York, N.Y., 10016, USA; 4 Department of Epidemiology of Mailman School of Public Health and Herbert Irving Comprehensive Cancer Center, Columbia University, New York, New York, USA

## Abstract

Prostate cancer is the most commonly diagnosed cancer in men and one of the leading causes of cancer deaths. There is strong genetic evidence indicating that a large proportion of prostate cancers are caused by heritable factors but the search for prostate cancer susceptibility genes has thus far remained elusive. TGFBR1*6A, a common hypomorphic variant of the type I Transforming Growth Factor Beta receptor, is emerging as a tumor susceptibility allele that predisposes to the development of breast, colon and ovarian cancer. The association with prostate cancer has not yet been explored. A total of 907 cases and controls from New York City were genotyped to test the hypothesis that TGFBR1*6A may contribute to the development of prostate cancer. TGFBR1*6A allelic frequency among cases (0.086) was slightly higher than among controls (0.080) but the differences in TGFBR1*6A genotype distribution between cases and controls did not reach statistical significance (p = 0.67). Our data suggest that TGFBR1*6A does not contribute to the development of prostate cancer.

## Background

Transforming Growth Factor Beta (TGF-ß) is one of the most potent inhibitor of cell growth [1]. Almost all cancer cells loose the ability to be growth inhibited by TGF-ß, which makes loss of TGF-ß growth inhibition a hallmark of cancer development [2]. TGFBR1*6A is a common variant of the type I TGF-ß receptor, *TGFBR1 *[3]. TGFBR1*6A (*6A) has a deletion of three GCG triplets coding for alanine within a nine alanine (9A) repeat sequence of *TGFBR1 *(*9A) exon 1, resulting in a six alanine (6A) repeat sequence. The 9-bp deletion that differentiates *6A from *9A is located within the predicted signal sequence cleavage region. *In vitro* studies have demonstrated that TGFBR1*6A responds less effectively than TGFBR1 to TGF-ß growth inhibitory signals [4,5]. The additional findings of an overrepresentation of TGFBR1*6A heterozygotes and homozygotes among patients with a diagnosis of cancer as compared with the general population led us to postulate that TGFBR1*6A might act as a tumor susceptibility allele [5]. Two recent meta-analyses show that TGFBR1*6A carriers may have an increased risk of breast, colon and ovarian cancer [6,7]. To test the hypothesis that TGFBR1*6A may contribute to the development of prostate cancer, we conducted a case control study of patients with biopsy verified prostate cancer cases and geographically and ethnic-status matched controls.

## Results

A total of 907 cases and controls were genotyped for TGFBR1*6A. The mean age of cases was significantly higher than controls (p < 0.01) but there were no differences in ethnic status between the two groups. There were 59 TGFBR1*6A heterozygotes and three TGFBR1*6A homozygotes among cases, 62 TGFBR1*6A heterozygotes and 1 TGFBR1*6A homozygote among controls. TGFBR1*6A allelic frequency among cases (0.086) was slightly higher than among controls (0.080) but the differences in TGFBR1*6A genotype distribution between cases and controls did not reach statistical significance (p = 0.67) (Table 1). Effect estimates from conditional logistic regression were similar (OR 1.01, 95% CI 0.29–3.52) to those from unconditional logistic regression (OR 0.96, 95% CI 0.56–1.64). Analyses restricted to subjects aged 40 years and above controlling age on a continuous scale produced essentially the same effect estimates (OR 0.94, 95% CI 0.56–1.60). To examine the possibility that TGFBR1*6A is associated with early onset prostate cancer, we determined the prostate cancer risk for individuals above and below the age of 55. Among younger patients with prostate cancer we found that 13 of 59 were TGFBR1*6A carriers yielding an allelic frequency of 0.119, one of the highest TGFBR1*6A allelic frequency ever reported. Only 45 of 367 controls in the same age range were TGFBR1*6A carriers yielding an allelic frequency of 0.063, which is similar to the TGFBR1*6A allelic frequency found among 3,451 healthy controls from Europe and the U.S. [7]. The association between carrier status of TGFBR1*6A and prostate cancer in younger age group was significant after adjustment for race (OR 2.13, 95% CI 1.06–4.27) but was not significant after adjustment for race and age strata within groups (OR 2.11, 95% CI 0.98–4.57) (Table 2). While in the older age group we did not observe a significant association either (OR 0.57, 95% CI 0.30–1.10), the test for multiplicative interaction between age and carrier status of TGFBR1*6A was significant (p = 0.01).

**Table 1:** Distribution of Age, Ethnicity, and *TGFBR1 *Genotypes and Adjusted Odds Ratios of Prostate Cancer by *TGFBR1 *Genotype Status

	Cases (N = 442)		Controls (N = 465)		P-value ^1^	Adjusted OR (95% CI)^2^
			
	N	%	N	%		
*TGFBR1 *genotype						
9A/9A	380	86.0	402	86.5	0.67	1.00 (ref)
9A/6A	59	13.4	62	13.3		0.96 (0.56–1.64)
6A/6A	3	0.6	1	0.2		
Age						
20–40	1	0.2	205	44.1	<0.01	
41–60	126	28.5	204	43.9		
61–80	308	69.7	55	11.8		
80+	7	1.6	1	0.2		
Race						
White	396	89.6	415	89.3	1.00	
Black	26	5.9	29	6.3		
Hispanic	8	1.8	8	1.7		
Asian	2	0.5	2	0.4		
Other	1	0.2	2	0.4		
Unknown	9	2.0	9	1.9		

^1^*p*-value for Chi-Square or Fisher's Exact Test (comparing proportions)

^2 ^OR was adjusted for age strata and race, based on dominant model.

**Table 2:** Adjusted Odds Ratios of prostate cancer by age groups (> 55, <= 55 years old)

Age group/ Genotypes	Cases N (% within age strata)	Controls N (% within age strata)	OR (95% CI)^1^	OR (95% CI)^2^	P for testing multiplicative interaction
Age = 55					
9A/9A	46 (78.0)	322 (87.7)	1.00	1.00	
9A/6A or 6A/6A	13 (22.0)	45 (12.3)	2.13 (1.06–4.27)	2.11 (0.98–4.57)	
Age > 55					0.01
9A/9A	334 (87.2)	80 (81.6)	1.00	1.00	
9A/6A or 6A/6A	49 (12.8)	18 (18.4)	0.64 (0.36–1.17)	0.57 (0.30–1.10)	

^1 ^OR was adjusted for race.

^2 ^OR was adjusted for race and age strata within age groups

## Discussion

Prostate cancer is the most common cancer and the second most common cause of cancer death among U.S. men [8]. A similar pattern is observed throughout the western world. There is strong epidemiologic evidence indicating that a large proportion of prostate cancers are caused by heritable factors. The most convincing data is a study of 44,788 Scandinavian twins showing that 42% of prostate cancer cancers may be caused by shared genes [9]. Despite intense efforts led by several research teams, the search for prostate cancer susceptibility genes has thus far remained elusive. Recent studies suggest that carriers of deleterious mutations of the *BRCA2 *gene have an increased prostate cancer risk [10]. However, given the low prevalence of deleterious *BRCA2 *mutations in the general population, it is unlikely to account for a significant proportion of prostate cancer cases. Approximately 14% of the general population carries at least one copy of TGFBR1*6A, which makes it the most common candidate tumor susceptibility allele reported to date. While there is growing evidence that TGFBR1*6A predisposes to the development of breast, colon and ovarian cancer, our data do not suggest that it predisposes to the development of prostate cancer. We have previously shown that TGFBR1*6A homozygotes have an O.R of 2.69 and 2.02 for ovarian and colon cancer, respectively. The present study has the power to detect an O.R. for prostate cancer of 1.70 or higher and therefore rules out a major association between TGFBR1*6A and prostate cancer. However, it does not exclude a smaller O.R., which might have clinical relevance given the high TGFBR1*6A allelic frequency in the general population. It is possible that age differences in cases and controls affected the allele frequencies observed. If the TGFBR1*6A allele predisposes to a lethal malignancy such as prostate cancer, however, its frequency could be higher, not lower, in a younger cohort. Thus, the younger mean age of controls could result in a bias toward the null hypothesis, resulting in a stronger association than that observed. The intriguing findings of a high TGFBR1*6A allelic frequency among prostate cancer cases diagnosed before the age of 55 have to be cautiously interpreted given the fact that this group only included 46 patients. We have previously shown that TGFBR1*6A is not associated with an increased risk of bladder cancer [6]. Our results suggest that the association between TGFBR1*6A and prostate cancer is at best very weak but further studies are needed to formally exclude an association with early onset prostate cancer.

## Methods

DNA was extracted from lymphocytes of blood specimens from 465 consecutive individuals diagnosed with adenocarcinoma of the prostate who received care at the outpatient urology clinic at Memorial Sloan-Kettering Cancer Center from April 2000 to September 2002. The blood samples were collected following completion of diagnostic studies. They were unselected for age or family history. Clinical and pathological records were reviewed to confirm the diagnosis of prostate cancer in all subjects. Once pathological diagnosis of prostate cancer was confirmed, the age of diagnosis was recorded, and all other identifying links were destroyed. The study design and anonymization method were approved by the Memorial Sloan-Kettering Cancer Center Institutional Review Board.

A population of 465 healthy male controls aged 20 to 87 years with well-defined ethnic background who had donated blood for various reasons (predominantly pre-natal screening for non-cancer disease) constituted the control group. Controls were matched to the cases on ethnicity and were from the same geographic locations as the prostate cancer cases. None of the controls had any personal history of cancer at the time of blood donation. This was ascertained by a questionnaire completed by each control. Exact age information was not available for 205 controls since it was not collected prospectively but the age range (20 to 40) was known. All personal identifiers were permanently removed from both cases and controls.

DNA was extracted by standard technique using the Qiagen DNA extraction kit. The PCR primers used were 5'-CCA CAG GCG GTG GCG GCG CGA TG-3' in the forward direction and 5'-CGT CGC CCC CGG GAF CAG CGC CGC-3' in the reverse direction. A standard solution was prepared using the Clontech Advantage^® ^GC rich kit (BD-Biosciences Clontech, Palo Alto, CA). The PCR reaction mixture included 20 ng of genomic DNA in a 10-µL reaction volume and the following concentration of other reagents: primers (0.25 µM each), 1X GC genomic PCR reaction buffer, 1.625 mM Mg^2+^, 0.2 mM dNTPs and 0.16 µL of Advantage-GC genomic polymerase mix. Polymerase chain reaction cycling conditions consisted of an initial denaturation period of 3 minutes at 94°C, then 35 cycles of denaturation for 30 seconds at 94°C and annealing/extension for 2 minutes at 72°C, followed by a final extension step of 5 minutes at 72°C. Quality controls were run on a 2% agarose gel. The ABI Prism 310 Genetic Analyzer (Applied Biosystems, Foster City, CA) was used for data acquisition. A peak at 115 base pairs corresponded to *TGFBR1 *allele, whereas a peak at 107 base pairs corresponded to the TGFBR1*6A variant. The rare equivocal results were confirmed by cloning of the PCR product followed by automated sequencing. Samples were read by two independent investigators unaware of the case /control status. Ten percent of samples were randomly selected and run for quality assurance. Concordance rate was 100%.

### Statistical analysis

Distributions of *TGFBR1 *genotypes, age, and ethnicity were compared between cases and controls using Fisher's exact tests. To test the hypothesis that the hypomorphic TGFBR1*6A gene is related to an increased prostate cancer risk, adjusted odds ratios of prostate cancer were estimated using both conditional and unconditional logistic regression models. Both models were run since the matched controls of cases with missing genotypes had to be excluded in the conditional models but could be included in the unconditional models. Adjusted odds ratios of prostate cancer were estimated comparing carriers of TGFBR1*6A versus non-carriers under dominant models. Potential confounders such as age (in four strata) and ethnicity were controlled in the analysis. Whether the effects of TGFBR1*6A on prostate cancer differ by age was evaluated by stratified analysis and tests for multiplicative interaction. A small p value indicates that interaction of age and gene is statistically significant on the multiplicative level. For the unconditional models, sensitivity analysis was conducted to evaluate the impact of the fact that the exact age of some controls with age 20–40 years is unknown (N = 126). With 442 cases and 465 controls, the power to detect an OR of 1.7 and 2 in the present study was 0.86 and 0.98, respectively, based on a two-tailed test at the 0.05 significance level.

## Authors' contributions

All authors made substantial contributions to this paper, including conceiving of the ideas, discussion and writing. All authors read and approved the final manuscript.
